# Itraconazole-Associated Fixed Drug Eruption in a Patient With Allergic Bronchopulmonary Aspergillosis: A Case Report and Review of the Literature

**DOI:** 10.7759/cureus.109661

**Published:** 2026-05-26

**Authors:** Reshma S Thomas, Girish Joseph, Neena Bhatti, Dinesh K Badyal

**Affiliations:** 1 Pharmacology, Christian Medical College and Hospital, Ludhiana, IND

**Keywords:** adverse drug reaction, anti-fungal therapy, cadr, fde, itraconazole, pharmacovigilance

## Abstract

Fixed drug eruption (FDE) is a well-recognized cutaneous adverse drug reaction (ADR) characterized by recurrent, sharply demarcated erythematous-to-violaceous plaques that heal with residual hyperpigmentation. Although commonly associated with several medications, FDE due to systemic azole antifungals is rare, with limited cases reported in the literature. This review summarizes recent evidence on azole antifungal-induced FDE, focusing on clinical features, mechanisms, and implications for practice. Available literature suggests that these reactions are mediated by a Type IVc delayed hypersensitivity response involving cytotoxic T lymphocytes. Reports also indicate potential intraclass cross-reactivity among triazole antifungals, which may complicate subsequent drug selection. Clinical presentations vary but consistently highlight the importance of early recognition and prompt withdrawal of the offending agent to prevent recurrence. Furthermore, underreporting in pharmacovigilance systems remains a challenge, limiting accurate estimation of incidence and risk factors. Despite its rarity, azole-induced FDE is clinically significant, emphasizing the need for heightened awareness, detailed drug history assessment, and systematic reporting to improve detection, understanding, and management of this uncommon ADR.

## Introduction

Adverse drug reactions (ADRs) are defined by the World Health Organization (WHO) as “a response to a medication that is noxious. unintended and occurs at doses normally used in man" [1]. Among ADRs, cutaneous adverse drug reactions (CADRs) are the most frequently encountered in clinical practice, ranging from benign exanthems to potentially life-threatening conditions such as Stevens-Johnson Syndrome (SJS) and toxic epidermal necrolysis (TEN) [2]. FDE is a clinically distinctive CADR defined by recurrent, sharply demarcated erythematous-to-violaceous plaques that arise at identical anatomical sites on each re-exposure to the offending drug, resolving with residual post-inflammatory hyperpigmentation [3]. A wide range of drugs has been implicated, including nonsteroidal anti-inflammatory drugs (NSAIDs), antibiotics, antiepileptics, and antimalarials; however, systemic azole antifungals are rarely reported causative agents [4,5].

Allergic bronchopulmonary aspergillosis (ABPA) is an immunologically mediated pulmonary disorder caused by hypersensitivity to Aspergillus fumigatus colonizing the airways, most commonly in patients with asthma or cystic fibrosis. Persistent fungal colonization triggers exaggerated immune responses characterized by elevated IgE levels, eosinophilic airway inflammation, recurrent pulmonary infiltrates, and progressive bronchiectasis. Corticosteroids remain the cornerstone of therapy because they suppress the underlying inflammatory response; however, systemic antifungal agents such as itraconazole are frequently used as steroid-sparing agents to reduce fungal burden, decrease antigenic stimulation, and minimize long-term corticosteroid exposure [6]. 

Itraconazole is a broad-spectrum triazole antifungal widely used for dermatophytosis, onychomycosis, systemic fungal infections, and as a steroid-sparing agent in ABPA [6]. Although generally well-tolerated, rare cutaneous reactions including FDE have been described [4,5]. According to VigiAccess pharmacovigilance data, approximately one-sixth of reported ADRs (4,699 cases) associated with itraconazole involve skin and subcutaneous tissue disorders; however, only 51 cases of fixed drug eruption (FDE) have been reported, underscoring the rarity of this reaction and the importance of documenting such cases to strengthen pharmacovigilance evidence and improve clinical recognition [7].

We report a probable case of itraconazole-associated FDE occurring in the specific clinical context of ABPA management and provide a focused review of the published literature on this rare entity, its pathophysiology, diagnostic approach, and management.

## Case presentation

A 67-year-old woman presented to the dermatology outpatient department with skin lesions over the anterior chest wall of six days’ duration. Her medical history was significant for bronchial asthma, hypertension, hypothyroidism, type 2 diabetes mellitus, obesity, and obstructive sleep apnea. The patient denied the use of any recent over-the-counter medications, herbal remedies, nutritional supplements, or alternative medicines before the onset of the lesions. Apart from her long-term prescribed medications for bronchial asthma, hypertension, hypothyroidism, and diabetes mellitus, no new drug had been introduced other than itraconazole. There was no personal or family history of atopic dermatitis, drug allergy, allergic skin disorders, or other hypersensitivity reactions. The patient also denied any prior episodes of eczema, urticaria, allergic rhinitis, food allergy, or previous cutaneous ADRs.

She had been admitted eight months earlier for type 2 respiratory failure requiring mechanical ventilation and intensive care management. Seven months post-discharge, she was evaluated for persistent respiratory symptoms and diagnosed with ABPA, supported by a markedly elevated total serum IgE of 2,385 IU/mL and Aspergillus-specific IgE of 16.7 kUA/L. Oral itraconazole 200 mg twice daily was initiated as adjunctive steroid-sparing therapy per established ABPA management guidelines. Relevant laboratory investigations supporting the diagnosis of ABPA are summarized in Table 1.

**Table 1:** Laboratory investigations supporting the diagnosis of allergic bronchopulmonary aspergillosis (ABPA). IgE, immunoglobulin E

Parameter	Patient value	Reference range/normal value
Total serum IgE	2,385 IU/mL	<100 IU/mL
*Aspergillus fumigatus*-specific IgE	16.7 kUA/L	<0.35 kUA/L

**Figure 1:**
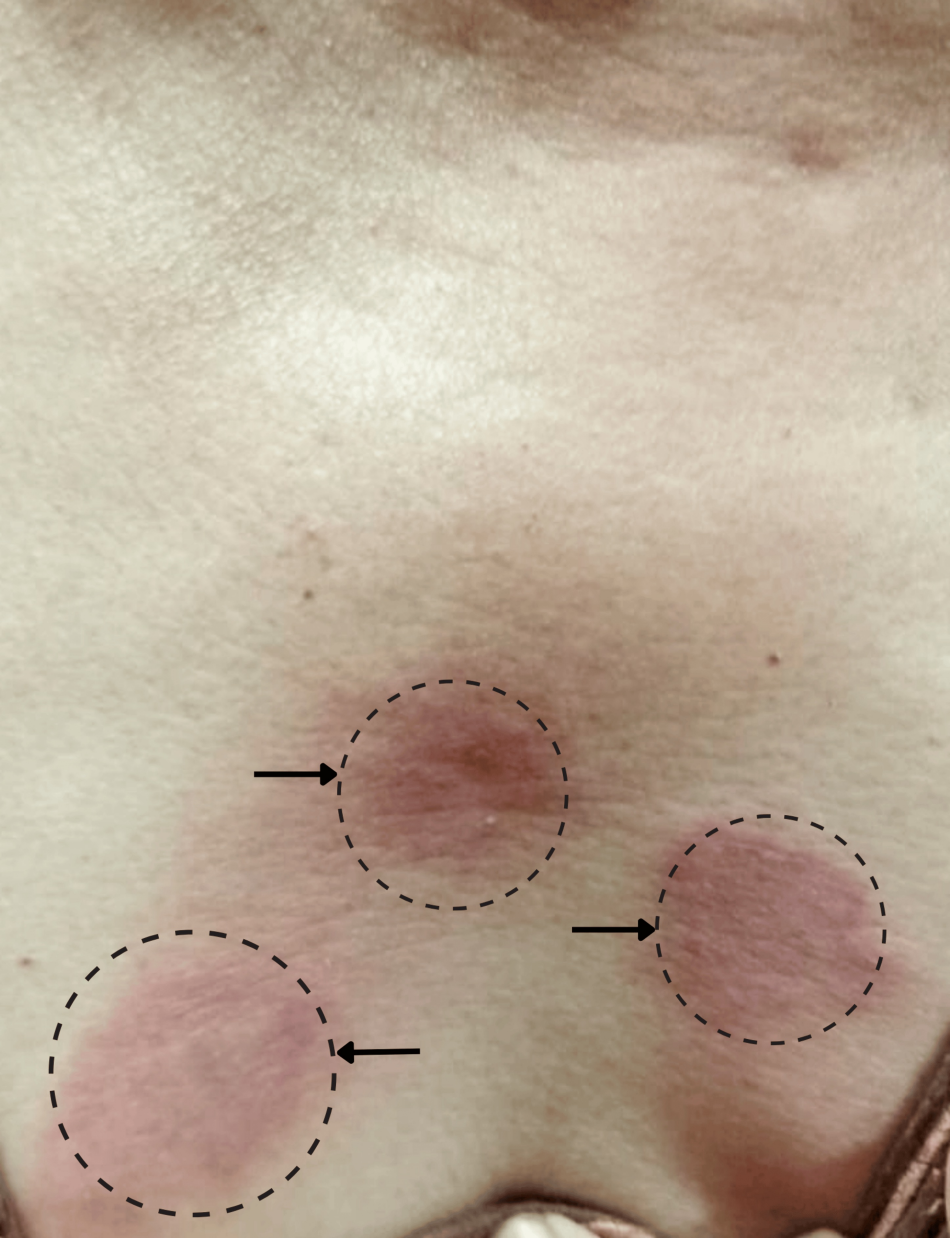
Active itraconazole-induced fixed drug eruption on the anterior chest wall. Multiple well-defined erythematous-to-violaceous plaques with central dusky discoloration and early erosion are present over the bilateral anterior chest wall in the supramammary region. The lesions are discrete and symmetrically distributed, consistent with the active phase of fixed drug eruption.

Following the second dose, the patient noticed multiple erythematous lesions over the anterior chest wall. Despite the appearance of lesions, she continued the medication until the fourth dose, following which the lesions became more prominent and medical attention was sought. Itraconazole was discontinued on the physician's advice. Mild symptomatic improvement was noted within four days of stopping the drug. She was subsequently referred to the dermatology outpatient department. A detailed description of the day of presentation is shown in Figure 1.

Cutaneous examination revealed multiple hyperpigmented-to-violaceous plaques with central erosions and superficial desquamation over the bilateral anterior chest wall, predominantly in the supramammary region, associated with pruritus. Hyperpigmented plaques with central healing and superficial desquamation represent the resolving phase of FDE following withdrawal of itraconazole. There was no similar involvement elsewhere on the body and no mucosal involvement. There was no history of previous similar reactions to any medication. Her other long-term medications for bronchial asthma, hypertension, and hypothyroidism had not changed; itraconazole was the sole new addition.

**Figure 2:**
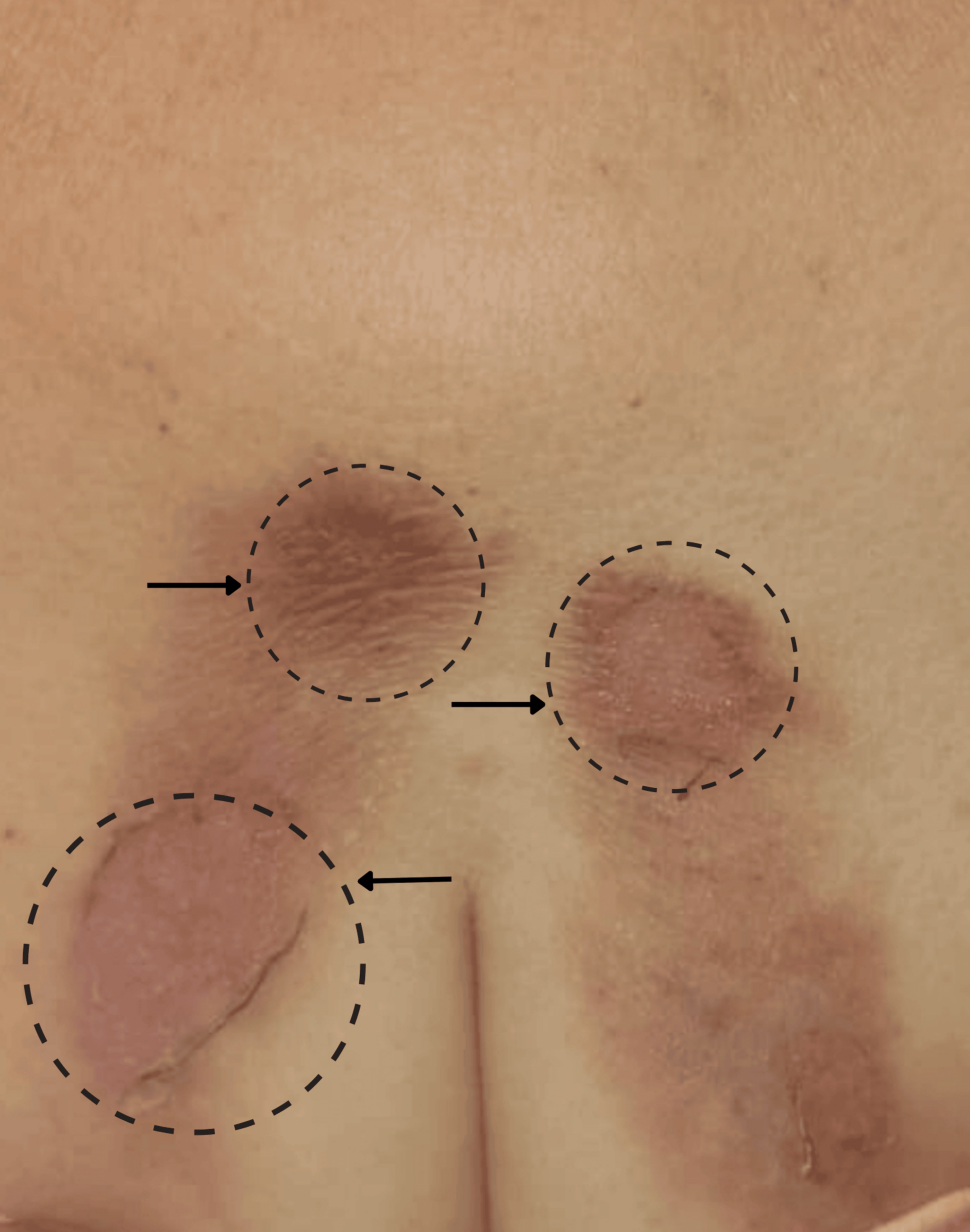
Resolving lesions with post-inflammatory hyperpigmentation. Well-demarcated hyperpigmented plaques with central healing and superficial desquamation are seen over the bilateral supramammary region, representing the resolving phase of fixed drug eruption following withdrawal of itraconazole.

Skin biopsy was not performed. Rechallenge was not undertaken due to ethical concerns and the risk of recurrence. The patient was managed with topical fusidic acid cream and oral levocetirizine 5 mg once daily for 10 days, resulting in gradual resolution of the active inflammation. The presentation on follow-up is shown in Figure 2.

ADR assessment

Structured pharmacovigilance assessment was performed using three validated instruments: the Naranjo Adverse Drug Reaction Probability Scale for causality, the Modified Hartwig and Siegel Severity Assessment Scale for severity grading, and the Schumock and Thornton Preventability Criteria for preventability [8-10]. The Naranjo Scale comprises 10 structured questions, each answered as “yes,” “no,” or “don’t know,” with weighted scores of +2, +1, 0, or −1. Total scores are categorized as definite (≥9), probable (5-8), possible (1-4), or doubtful (≤0) [8]. The detailed Naranjo scoring is shown in Table 2.

**Table 2:** Naranjo Adverse Drug Reaction Probability Scale scoring.

Question	Response	Score
Previous conclusive reports of this reaction?	Yes	+1
Did the adverse event appear after the suspected drug was administered?	Yes	+2
Did the reaction improve after discontinuation of the drug?	Yes	+1
Did the reaction reappear on re-administration?	Not done	0
Are there alternative causes that could explain the reaction?	No	+2
Did the reaction reappear with the placebo?	Not applicable	0
Was the drug detected in toxic concentrations?	Not done	0
Was the reaction more severe with an increased dose or less severe with a decreased dose?	Yes	+1
Did the patient have a similar reaction to the same or related drugs previously?	No	0
Was the adverse event confirmed by objective evidence?	No biopsy/patch test performed	0

A summary of the ADR assessment is described in Table 3. 

**Table 3:** Summary of ADR assessment using standardized pharmacovigilance tools. ADR, adverse drug reaction; ABPA, allergic bronchopulmonary aspergillosis

Assessment tool	Criteria applied	Result/Interpretation
Naranjo Adverse Drug Reaction Probability Scale [8]	Temporal relationship between itraconazole initiation and eruption onset; dose-dependent worsening; clinical improvement on dechallenge; absence of alternative causative agents; prior case reports in the literature	Score = 7 → Probable Adverse Drug Reaction
Modified Hartwig and Siegel Severity Assessment Scale [9]	Drug discontinuation required; active medical treatment (oral antihistamine, topical antibiotic) necessary; no hospitalization or permanent harm	Level 3 → Moderate Severity Adverse Drug Reaction
Schumock and Thornton Preventability Criteria [10]	Although the initial reaction was not reasonably foreseeable in the absence of prior azole hypersensitivity history, future recurrence was considered probably preventable through prompt identification, permanent avoidance of itraconazole, patient counselling, and documentation regarding possible triazole cross-reactivity.	Yes → Probably Preventable Adverse Drug Reaction

Written informed consent was obtained from the patient for publication of this case report, including the use of clinical details and the image of the patient. All efforts have been made to ensure patient anonymity, and no identifiable information has been disclosed. The case was documented as part of routine pharmacovigilance activity, and institutional guidelines for reporting ADRs were followed. This case was reported to the nearest ADR monitoring center, with the Worldwide Unique ID number being IN-IPC-301255038. 

## Discussion

FDE is a clinically distinctive cutaneous ADR characterized by recurrent, sharply demarcated erythematous-to-violaceous plaques that recur at identical anatomical sites upon re-exposure to the offending drug and heal with residual post-inflammatory hyperpigmentation [3]. Although a broad spectrum of medications, including NSAIDs, antibiotics, antiepileptics, and antimalarials, have been implicated, systemic azole antifungals remain a rare cause of FDE [4,5,11,12]. According to VigiAccess pharmacovigilance data, approximately one-sixth of ADRs reported with itraconazole involve skin and subcutaneous tissue disorders; however, only 51 reported cases are categorized as FDE, highlighting the rarity of this reaction and emphasizing the importance of documenting such cases to strengthen pharmacovigilance evidence and improve clinical recognition.

FDE was first described by Bourns in 1889 and remains one of the most recognizable forms of cutaneous ADR because of its characteristic site-specific recurrence pattern [3]. It may occur in individuals of any age but is more frequently reported among young and middle-aged adults [11]. The pathophysiology is classified as a Type IVc delayed hypersensitivity reaction mediated predominantly by CD8+ cytotoxic T lymphocytes [13]. During initial drug exposure, drug-specific CD8+ T cells become sensitized and localize within the epidermis as tissue-resident memory T cells (Trm), where they persist in a quiescent state under the influence of cytokines such as interleukin-15 [14]. Upon re-exposure to the culprit drug, these resident memory cells are rapidly reactivated and release inflammatory mediators, including interferon-gamma, perforin, granzyme B, and tumor necrosis factor-alpha, leading to keratinocyte apoptosis and melanocyte damage [13,14]. Pigmentary incontinence secondary to melanocyte injury produces the hallmark residual hyperpigmentation seen after lesion resolution.

For azole antifungals specifically, the precise mechanism of sensitization remains incompletely understood. Itraconazole or its metabolites may function as haptens or pro-haptens by binding to carrier proteins and generating immunogenic peptide complexes, or may activate immune pathways through the pharmacological interaction (p-i) mechanism without prior haptenization [15]. The shared 1,2,4-triazole ring structure among itraconazole, fluconazole, voriconazole, and posaconazole has been proposed as a possible explanation for intraclass cross-reactivity observed in certain patients [4].

Clinically, FDE typically manifests as one or more sharply demarcated erythematous or dusky violaceous plaques associated with burning sensation or pruritus [3,12]. Lesions may occasionally progress to blistering or bullous forms, and severe generalized bullous FDE (GBFDE) may mimic Stevens-Johnson syndrome or toxic epidermal necrolysis [12]. Residual post-inflammatory hyperpigmentation following lesion resolution remains a diagnostic hallmark and may persist for weeks to months [3]. Commonly affected sites include the lips, face, genitalia, hands, and trunk, although virtually any cutaneous or mucosal site may be involved [11,12]. Histopathological examination, when performed, typically demonstrates vacuolar interface dermatitis with dyskeratotic keratinocytes, superficial lichenoid lymphocytic infiltrates predominantly composed of CD8+ T cells, and pigmentary incontinence with dermal melanophages [12]. Although not pathognomonic, these findings strongly support the diagnosis when correlated with clinical history.

Itraconazole is a broad-spectrum triazole antifungal widely used in dermatophytosis, onychomycosis, systemic fungal infections, and as a steroid-sparing agent in ABPA [6]. Its antifungal activity results from inhibition of fungal cytochrome P450-dependent 14α-lanosterol demethylase, thereby disrupting ergosterol synthesis within fungal cell membranes [6]. The drug undergoes extensive hepatic metabolism through CYP3A4 and is associated with significant drug-drug interaction potential. Common adverse effects include gastrointestinal intolerance and headache, while serious reactions such as hepatotoxicity, congestive heart failure, QT prolongation, Stevens-Johnson syndrome, toxic epidermal necrolysis, and FDE are uncommon [16]. Published literature regarding itraconazole-induced FDE remains limited to isolated case reports.

A review of the available literature identified only a few documented cases of itraconazole-associated FDE, which are summarized in Table 4.

**Table 4:** Summary of published cases of itraconazole-associated fixed drug eruption. M, male; F, female; ABPA, allergic bronchopulmonary aspergillosis

Author/year	Age/sex	Indication	Onset	Site(s)	Outcome/notes
Gupta and Thami, 2008 [4]	Adult/M	Dermatophytosis	After the second dose	Genitalia, lips	Resolved on withdrawal; cross-reactivity with fluconazole confirmed on re-exposure
Guliani and Chauhan, 2019 [5]	Adult/M	Onychomycosis	After the third dose	Trunk, extremities	Resolved after drug withdrawal; managed with antihistamines
Sil and Das, 2020 [17]	Adult/F	Dermatophytosis	Within 48 hours of initiation	Face, hands	Positive patch test at affected sites; resolved with topical corticosteroids
Present case, 2025	67 years/F	ABPA	After the second dose	Bilateral anterior chest wall (supramammary)	Naranjo score 6 (probable); resolved with levocetirizine and topical fusidic acid; no prior drug reactions

Across reported cases, itraconazole-associated FDE consistently develops early during therapy, generally within the first few doses, suggesting either rapid immune activation or prior sensitization [4,5,17]. Lesion distribution has varied considerably among cases, involving genitalia, lips, trunk, extremities, face, and chest wall, consistent with the concept that any site containing resident memory T cells may become a focus of recurrent inflammation [14]. Importantly, all published cases demonstrated improvement following prompt withdrawal of itraconazole without requiring hospitalization. Causality assessments in most reports fell within the “probable” category because oral rechallenge, although considered the diagnostic gold standard, was avoided for ethical reasons due to the risk of recurrence or progression to generalized disease [12].

Potential intraclass cross-reactivity among triazole antifungals has important therapeutic implications. Gupta and Thami demonstrated recurrence of lesions following inadvertent administration of fluconazole in a patient previously sensitized to itraconazole, establishing that cross-reactivity may occur at least in certain individuals [4]. Consequently, patients diagnosed with itraconazole-induced FDE should be counselled regarding possible reactions to other triazoles. Where feasible, alternative antifungal classes such as amphotericin B or echinocandins may be considered. If use of another triazole becomes unavoidable, cautious supervised administration with informed consent may be necessary [4,18].

Diagnosis of FDE remains primarily clinical and relies on recognition of characteristic morphology, site-specific recurrence, residual hyperpigmentation, and a clear temporal relationship with drug exposure [3,12]. Detailed drug history, including prescription medications, over-the-counter agents, herbal remedies, and supplements, remains essential [3]. Patch testing at previously affected sites performed several weeks after lesion resolution offers a relatively safe confirmatory modality and demonstrates greater sensitivity than testing uninvolved skin [12]. Histopathology may assist in atypical cases or medicolegal documentation, but it is not mandatory. Oral rechallenge remains the definitive confirmatory test but is generally discouraged in routine practice because of the potential for severe recurrence or GBFDE [12]. In the present case, biopsy, patch testing, and rechallenge were not performed because the clinical presentation and temporal association were strongly suggestive, and rechallenge posed ethical concerns.

The diagnosis in the present case was therefore established primarily on clinical grounds by the treating dermatologist based on the characteristic morphology of the lesions, residual post-inflammatory hyperpigmentation, temporal association with itraconazole administration, worsening with continued exposure, and improvement following drug withdrawal. Additional confirmatory investigations, such as lymphocyte transformation testing (LTT), CD4+ T-cell proliferation assays, and histopathological examination, were not performed because of limited availability and the sufficiently characteristic clinical correlation. Although LTT has been reported to demonstrate sensitivity and specificity of approximately 65.1% and 96.5%, respectively, in mild-to-moderate drug hypersensitivity reactions, its routine use in FDE remains limited due to restricted accessibility and lack of standardization for azole antifungal-associated reactions.

The present case is particularly noteworthy because itraconazole was administered for the management of ABPA, where it serves as an established steroid-sparing agent [6]. Development of FDE in this setting carries important therapeutic implications, as premature discontinuation of itraconazole may compromise disease control and increase corticosteroid burden. Voriconazole and other antifungals may be considered as alternatives, although each possesses its own adverse effect profile [6]. This case, therefore, highlights the importance of dermatological surveillance and early recognition of cutaneous adverse reactions in patients receiving prolonged azole therapy, especially those with multiple comorbidities.

This case demonstrated several features distinct from previously published reports. To the best of our knowledge, this is the first reported case of itraconazole-induced FDE occurring in the context of ABPA management. Furthermore, bilateral supramammary chest wall involvement has not previously been described. The dose-dependent progression of lesions, with onset after the second dose and worsening after continued administration up to the fourth dose, is consistent with progressive activation of resident memory CD8+ T cells [14]. Absence of prior drug hypersensitivity reactions suggests sensitization specific to itraconazole rather than generalized drug hypersensitivity predisposition.

Structured pharmacovigilance assessment further strengthened the likelihood of a causal association in this case. The Naranjo Adverse Drug Reaction Probability Scale yielded a score of 6, categorizing the reaction as *probable *[8]. The Modified Hartwig and Siegel Scale classified the reaction as moderate in severity, while the Schumock and Thornton criteria identified the event as probably preventable [9,10]. These findings emphasize the importance of obtaining detailed drug allergy histories and maintaining awareness regarding rare but clinically significant cutaneous reactions associated with azole antifungals.

Reporting rare ADRs, such as itraconazole-associated FDE, remains critically important for pharmacovigilance systems, including WHO VigiAccess and the Pharmacovigilance Programme of India (PvPI) [19]. Rare cutaneous ADRs are frequently underrecognized and underreported, limiting accurate assessment of incidence, risk factors, and cross-reactivity patterns. Each additional published case contributes incrementally to defining the clinical spectrum and safety profile of itraconazole-induced FDE and may ultimately influence prescribing recommendations and drug safety labelling.

Overall, this case expands the limited global literature on itraconazole-associated FDE and reinforces the importance of early recognition, prompt withdrawal of the offending drug, patient counselling regarding possible triazole cross-reactivity, and systematic pharmacovigilance reporting.

Limitations

This case report and review have several inherent limitations. As a single-patient observation, the findings cannot be generalized, and population-level incidence or risk estimates cannot be derived. The diagnosis was established on clinical and temporal grounds without histopathological confirmation, which limits diagnostic certainty. Patch testing at previously affected sites was not performed, and oral rechallenge was ethically contraindicated; the absence of rechallenge data precluded a definite Naranjo score. Long-term follow-up was limited, restricting assessment of hyperpigmentation persistence or recurrence. Finally, the paucity of published itraconazole-induced FDE cases limits the strength of conclusions regarding incidence, risk factors, and long-term outcomes.

Future directions

Future research should focus on multicenter prospective registries to systematically document azole antifungal-induced FDE, enabling better characterization of incidence, clinical patterns, and drug-specific risk. Immunological studies exploring the precise role of tissue-resident memory T cells in itraconazole sensitization, and the structural determinants of intraclass triazole cross-reactivity, may clarify pathogenesis and guide safer drug substitution. The diagnostic value of patch testing and in-vitro assays (e.g., lymphocyte transformation tests) warrants prospective evaluation. Integration of pharmacogenomic approaches may ultimately enable the identification of susceptible individuals and support personalized antifungal prescribing. Strengthening pharmacovigilance reporting culture among clinicians across specialties is essential to generate the robust safety data needed to inform clinical guidelines.

## Conclusions

Itraconazole-associated FDE is a rare but clinically significant cutaneous ADR. This case, occurring in a patient receiving itraconazole as adjunctive therapy for ABPA, adds to the limited published literature on azole antifungal-associated FDE. The diagnosis was established primarily on clinical grounds based on characteristic lesion morphology, temporal association with itraconazole exposure, progression following continued administration, and improvement after drug withdrawal.

The patient demonstrated gradual resolution of lesions following discontinuation of itraconazole and symptomatic treatment with oral levocetirizine and topical fusidic acid. Alternative management strategies for ABPA were subsequently considered by the treating clinical team. Although intraclass cross-reactivity among triazole antifungals has been reported in the literature, it remains a theoretical concern in this case and warrants caution when considering future azole therapy.

Structured pharmacovigilance assessment classified the reaction as a probable ADR with moderate severity. The ADR was also reported through the institutional pharmacovigilance reporting system to contribute to ongoing drug safety surveillance. This case highlights the importance of early recognition of cutaneous adverse reactions, prompt withdrawal of the suspected drug, careful documentation, and continued pharmacovigilance reporting to improve understanding of rare itraconazole-associated adverse reactions.
